# Interaction network effects on position- and velocity-based models of collective motion

**DOI:** 10.1098/rsif.2020.0165

**Published:** 2020-08-19

**Authors:** Ali Emre Turgut, İhsan Caner Boz, İlkin Ege Okay, Eliseo Ferrante, Cristián Huepe

**Affiliations:** 1 Department of Mechanical Engineering, Middle East Technical University, Ankara, Turkey; 2 Department of Computer Science, Vrij Universiteit Amsterdam, De Boelelaan 1105, 1081 HV, Amsterdam, The Netherlands; 3 CHuepe Labs, 2713 West Haddon Ave #1, Chicago, IL 60622, USA; 4 Northwestern Institute on Complex Systems and ESAM, Northwestern University, Evanston, IL 60208, USA

**Keywords:** collective motion, Vicsek model, Active-Elastic model, interaction topology, complex networks, order–disorder transition

## Abstract

We study how the structure of the interaction network affects self-organized collective motion in two minimal models of self-propelled agents: the Vicsek model and the Active-Elastic (AE) model. We perform simulations with topologies that interpolate between a nearest-neighbour network and random networks with different degree distributions to analyse the relationship between the interaction topology and the resilience to noise of the ordered state. For the Vicsek case, we find that a higher fraction of random connections with homogeneous or power-law degree distribution increases the critical noise, and thus the resilience to noise, as expected due to small-world effects. Surprisingly, for the AE model, a higher fraction of random links with power-law degree distribution can decrease this resilience, despite most links being long-range. We explain this effect through a simple mechanical analogy, arguing that the larger presence of agents with few connections contributes localized low-energy modes that are easily excited by noise, thus hindering the collective dynamics. These results demonstrate the strong effects of the interaction topology on self-organization. Our work suggests potential roles of the interaction network structure in biological collective behaviour and could also help improve decentralized swarm robotics control and other distributed consensus systems.

## 1. Introduction

In the study of complex systems, the dynamics of multiple interacting components is typically analysed using one of two different modelling approaches: agent-based or network-based. If the components can be characterized as particles moving in a physical or abstract space and interactions depend on their positions, the system is best described using agent-based approaches. Alternatively, if the components can be characterized as nodes in a network, with internal states that evolve by interacting through their connections, it is best described using network-based approaches. Both types of models have been widely used to analyse the self-organized collective dynamics observed in a broad range of complex systems.

A simple form of self-organization (that can be observed in, both, agent-based and network-based systems) consists of individuals reaching the same *consensus* state, despite having no central control or global exchange of information. A well-known example is the dynamics followed by *distributed consensus* algorithms [1–3], where components reach global consensus on the values of their corresponding variables by communicating only with direct neighbours. Specifically, in a *distributed average consensus* protocol on networks [2], a variable associated to each node is iteratively replaced by the average of the variables of the nodes directly connected to it, a process that is repeated until they all converge to a common value. An interesting question in this kind of system is how these consensus dynamics depend on network topology, noise and local failures. This is an area of active research with multiple applications [4].

Distributed average consensus algorithms have been used in the decentralized control of groups of mobile autonomous agents, such as robot swarms [5,6], and to model the collective dynamics of groups of biological agents, such as bird flocks, fish schools or herds of quadrupeds [7–9]. In all these cases, the components are self-propelled agents that must reach decentralized speed and heading consensus in order to achieve collective motion. The connection between distributed average consensus and collective motion is apparent in one of the most common flocking algorithms, the Vicsek model [10], where each agent iteratively replaces its current heading by the local average heading of all nearby neighbours (including itself). In this model, the interaction network is typically dynamic: the focal agent interacts with all agents within a given range. Thus, the neighbours of each agent can change over time.

Although most collective motion algorithms are based on underlying distributed consensus dynamics, a different mechanism for achieving self-organization was unveiled in the recently introduced Active-Elastic (AE) model, where agents interact by exchanging only their relative positions [11,12]. In this model, each agent interacts with a fixed set of neighbours (determined a priori) throughout the simulation. The interaction topology is therefore fixed.

In this model, collective motion cannot result from a standard distributed consensus mechanism, since the speed and heading angles are never communicated between agents. Instead, collective motion is achieved through the damping of high-energy elastic modes (corresponding to short wavelengths and thus to small scales of coherent motion) and the focusing of self-propulsion energy into low-energy modes (corresponding to long wavelengths and thus to large scales of coherent motion).

In this paper, we explore the relationship between the interaction topology and self-organization of two different types of models of collective motion: one velocity-based, represented by the Vicsek model, and one position-based, represented by the AE model. We study their resilience to noise for different fixed interaction networks that are not constrained only to nearest neighbours, but can also contain interactions with randomly chosen agents, which are typically long-range since these can be located anywhere in the system. More specifically, we compare the critical noise of the order–disorder transition when agents interact through combinations of three different types of connectivity: (i) nearest-neighbour (NN) networks, (ii) Erdös–Rényi (ER) random networks, and (iii) scale-free (SF) random networks. We show that the interaction topology affects both models strongly but in very different ways and that, surprisingly, in some cases long-range SF connections can hinder the collective dynamics.

Our results could have several applications, both in the study of biological groups displaying collective motion and in the design of engineered collective dynamics for robot swarms. In biological systems, our work could help understand the connection between complex interaction networks and emergent collective behaviour. For example, it has been shown in recent experimental studies that certain fish schools have non-trivial interaction networks (resulting from their visual, line-of-sight based interactions) that affect their group dynamics [13]. It could also be relevant for the ongoing discussion on whether interactions are distance-dependent or topological in bird flocks [14–16]. In engineered systems, our work could help better exploit the effects of the communication network in robot swarms [17–19]. It could even provide insights beyond collective robotics, for the design of other distributed systems that exploit, for example, scale-invariant topologies [20] to achieve consensus on wireless sensor networks with reduced energy consumption [21–23] or with increased robustness to failure [23,24].

The paper is organized as follows. In §2, we describe the alignment-based Vicsek model and the position-based AE model used in this work. Section 3 details the different interaction topologies that we consider and how we interpolate between them. Section 4 presents our simulations and results, showing how the order–disorder transition depends on the interaction topology in both models. The discussion in §5 provides a heuristic explanation of our results. Finally, §6 gives our conclusion.

## 2. Collective motion models

In this section, we will describe the two models of collective motion considered in this paper: the Vicsek model [10] and the AE model [11]. The Vicsek model assumes that each agent can only know the orientation of its neighbours and that interactions are purely based on alignment. Conversely, the AE model assumes that each agent can only know the position of its neighbours and that interactions are attraction/repulsion-based. We selected these two models because they are archetypal examples of two different self-organizing mechanisms that can lead to collective motion. More realistic models often combine both types of interactions, as for example in [25].

The main difference in our implementation of these models is that here we will consider an interaction network that is not only restricted to nearest neighbours and that is fixed throughout the simulation. In the case of the AE model, the original algorithm already considered a fixed interaction network, so the only change is to include long-range connections. In the case of the Vicsek model, however, its original formulation establishes interactions through a proximity network that links all agents within a given distance of each other at each moment. Here we replace this evolving network by a fixed network with given topology, to study how the connectivity structure affects self-organization. We will refer to this version of the Vicsek model (where agents interact through a fixed arbitrary network) as the Vicsek-Network (VN) model. Note that the same Vicsek algorithm implementation on a network was introduced in [26], where it was referred to as the Vectorial Network model. The topologies that were considered there were simpler, however, since the main purpose of that study was to perform analytical calculations. Each agent thus received information from a fixed number of nodes through directed connections, with a constant fraction of these being first neighbours and the rest selected at random. The undirected complex topologies considered here were thus not part of that original Vectorial Network model formulation.

### 2.1. The Vicsek-Network model

As stated above, in the VN model the interacting agents are fixed and predetermined by the interaction network topology. This implies that it is not necessary to keep track of agent positions in our simulations, since these only serve to establish which agents are close enough to interact in the original Vicsek model [10]. We therefore implemented a VN model where the heading angles *θ*_*i*_(*t*) fully define the state of all agents at time *t*. The time step of our VN model is thus simply given by

2.1 $$\theta_{i}(t+1)=\text{Angle}\left[\sum_{\;j\in S_{i}}{\hat{n}}_{j}(t)\right]+\eta \;\xi_{i}(t).$$

Here, ${\hat{n}}_{j}(t)$ is a unit vector pointing in the heading direction of agent *j* at time *t*, the set *S*_*i*_ contains the indexes of all agents that interact with agent *i* (thus defining the interaction network), the function Angle [ · ] gives the angle of the vector in its argument, *ξ*_*i*_(*t*) is a random variable uniformly distributed between − 1/2 and 1/2, and the control parameter *η* determines the noise intensity level (with *η* = 0 for no noise and *η* = 2*π* for fully random motion).

### 2.2. The Active-Elastic model

We implemented a version of the AE model very similar to the original one introduced in [11,12]. As in the original version, agents move on a two-dimensional plane and are connected by an interaction network of linear spring-like forces. The position ***x***_*i*_ and orientation *θ*_*i*_ of each agent *i* satisfy the following two overdamped equations of motion

2.2 $${\dot{x}}_{i}(t)=v_{0}\;{\hat{n}}_{i}(t)+\alpha [F_{i}(t)\cdot {\hat{n}}_{i}(t)]\;{\hat{n}}_{i}(t)$$

and

2.3 $${\dot{\theta }}_{i}(t)=\beta [F_{i}(t)\cdot {\hat{n}}_{i}^{⊥}(t)]+\eta \;\xi_{i}(t).$$

Here, ${\hat{n}}_{i}(t)$ is a unit vector that points in the heading direction of agent *i* at time *t* (as in the VN model) and ${\hat{n}}_{i}^{⊥}(t)$ is a unit vector pointing perpendicular to it. The value of *v*_0_ determines the preferred self-propulsion speed, while *α* and *β* are the coupling coefficients that relate the interaction force ***F***_*i*_(*t*) to the linear and angular speed of each agent, respectively. As in the VN case, noise is introduced through *ξ*_*i*_(*t*), a random variable uniformly distributed between − 1/2 and 1/2, and noise intensity is controlled by *η*. Note that we chose this noise formulation to be consistent with the original Vicsek and AE models [10,11], but that the results presented in this paper should not significantly depend on the specific way that noise is introduced.

In this version of the AE model, we define ***F***_*i*_ as the following sum of all elastic forces acting over agent *i*:

2.4 $$F_{i}(t)=\kappa \sum_{\;j\in S_{i}}(∥r_{ij}(t)∥-l_{ij})\frac{r_{ij}(t)}{∥r_{ij}(t)∥},$$

where ***r***_*ij*_(*t*) = ***x***_*j*_(*t*) − ***x***_*i*_(*t*) and *κ* is the ‘spring constant’. Each term in the sum corresponds to the force exerted between agents *i* and *j* by a linear spring with natural length *l*_*ij*_. As in the VN model, each set *S*_*i*_ contains the *j* indexes of all agents linked to agent *i* in the fixed interaction topology considered. A difference with the original AE model is that the interaction strength *κ* is the same for any natural length, while in its original version the spring constant was inversely proportional to *l*_*ij*_. Here, however, we are interested in having the same interaction strength, regardless of distance, in order to compare the VN and AE models on equal footing. In our preliminary analyses, we also checked that the same qualitative effects described below are observed when simulating the original AE model.

**Figure 1.**
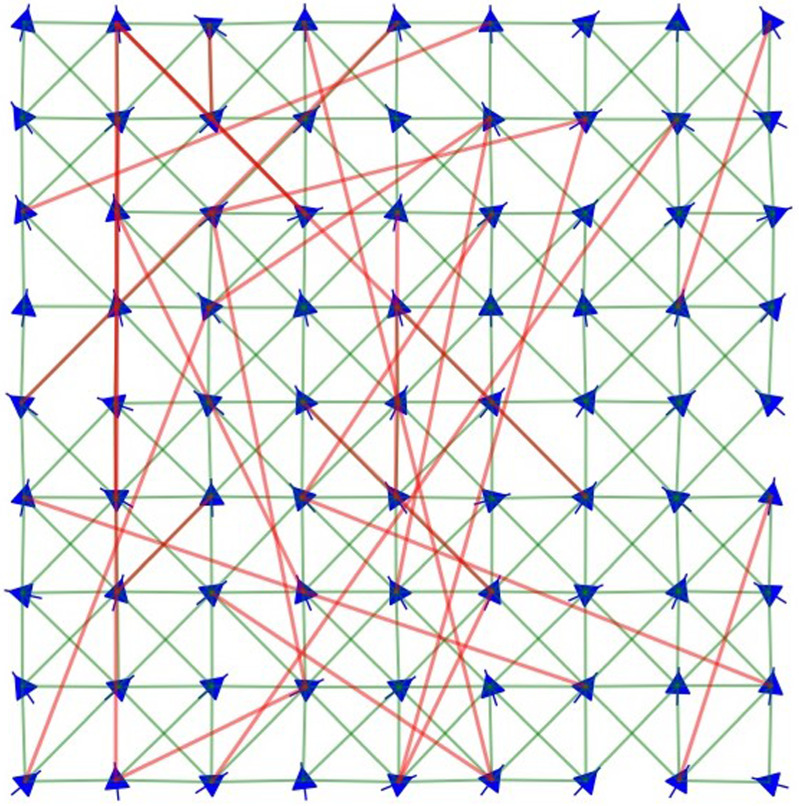
Simulation snapshot of a 9 × 9 agent system with its corresponding connectivity diagram. The blue arrows show the positions and orientations of the agents and the lines represent their interactions. The displayed interaction topology is the superposition of a NN network (green links) and a homogeneous ER random network (red links). The displayed state presents partial alignment (with agents mostly heading upwards) and has stiff spring-like forces, so the regular square lattice of agent positions is only slightly deformed.

In practice, our simulations are performed as follows. Once all agents are placed in a rectangular lattice (as in figure 1) and the network topology is defined, the *l*_*ij*_ values are set to *l*_*ij*_ = ||***x***_*j*_(0) − ***x***_*i*_(0)||, corresponding to the initial distances between agents *i* and *j*. This way, we can be certain that there are no elastic forces at *t* = 0 and that all stresses will be produced later by the self-propulsion dynamics. We then integrate equations (2.2) and (2.3) in time by implementing a standard Forward Euler Method, as detailed in [12]. Note that, in contrast to our VN simulations, spatial positions must be tracked here because they determine the distance, and thus the force, between each pair of interacting agents.

Finally, it is important to point out that although we used the same variable *η* to label the noise intensity in the VN and AE models, the magnitude of this parameter cannot be compared between them, since *η* controls the noise applied at each time step in the former, whereas it determines the noise level in a differential equation in the latter. An interesting possibility to overcome this would be to compute the effective temperature suggested in [27] for both models and then compare their critical values. However, a clear interpretation of this temperature was only provided in this reference for the VN case. Its exact meaning when comparing the critical noise of two models with very different interactions, self-organizing mechanisms and time step settings, is therefore unclear. Using this approach to quantitatively compare the critical noise values of both models would thus require to first examine the significance of this effective temperature for the AE model, which is an interesting analysis but is beyond the scope of this work. This does not limit our results, however, since we are only interested here in comparing how the noise effects depend on the topology within each model, and not in comparing their actual values between models.

## 3. Interaction networks

**Figure 2.**
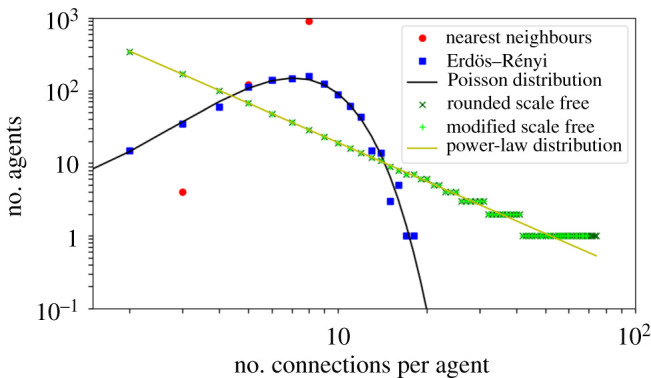
Degree distributions of the three types of interaction networks implemented in this paper (nearest neighbour, Erdös–Rényi and scale-free) for a 9 × 9 = 81 agent system with 272 connections (as in figure 1). Each plot shows the number of agents that have a given number of interactions with other agents. In the nearest-neighbour network (red dots), connections are determined by the number of immediate neighbours in a square lattice, here three, five or eight for agents in the corners, sides or bulk, respectively. In the Erdös–Rényi network (blue squares), the degree distribution must be Poissonian, as approximated by the implemented case displayed. Finally, in scale-free networks (× and + signs) the degree distribution must follow a power-law, here well approximated by the *modified scale free* connectivity case (which was generated by manually correcting the *rounded scale free* case), despite the finite and discrete nature of the system.

We will now describe the main interaction topologies used in this work and how we interpolated between them. We will detail below three different types of networks: (1) NN networks [28,29], (2) random ER networks [30] and (3) random SF networks [31]. Each type has a distinct degree distribution, as shown in the plots of the number of nodes with a given number of connections presented in figure 2. These networks will be later combined to study how our VN and AE simulations change when interacting through different superpositions of their topologies.

In order to compare the dynamics resulting from various interaction topologies under equivalent conditions, we must keep constant the total number of nodes *N* and the total number of links *K* over all connectivity structures. This also ensures that the average number of connections per node *K*/*N* is constant, although other statistical quantities may change as a result of the different degree distributions of the networks. We will detail below how *N*, *K*, and the system size are related in our NN networks and how we prepared ER and SF network topologies with predetermined *N* and *K* values.

### 3.1. Nearest-neighbour networks

To build our NN networks, we first placed the agents in a *L* × *L* square lattice configuration with unit distance between vertical and horizontal neighbours. This determines the initial positions of *N* = *L* × *L* agents in space, as presented in figure 1 for *L* = 9 and *N* = 81. Each agent is then connected with its horizontal, vertical and diagonal first neighbours. The agents in the bulk will thus have eight connections, those on the sides will have five connections and the four corner agents will have three connections. The resulting total number of links will be $K=2(L-1)(2L-1)=2(\sqrt{N}-1)(2\sqrt{N}-1)$ and is uniquely determined by the system size.

We note that the NN topology is the only one, of the three considered in this work, that is purely based on local connectivity. As in most standard models of collective motion, its structure is therefore determined by the agents’ positions in space. In this case, their positions at *t* = 0 determine the fixed interaction network at all times, instead of having a changing proximity interaction network that is redefined at every *t*.

### 3.2. Erdös–Rényi networks

We are interested in considering ER random networks that match the *N* and *K* values set by the system size for our NN networks. These will be generated using the following algorithm.

We start by defining *N* nodes with no connections. We then randomly select a pair of nodes (*i*, *j*) and connect them, unless we have *i* = *j* or they were already linked. We repeat this process until *K* links are established and then verify that the resulting network is connected. If so, we have generated our random ER network with *N* nodes and *K* connections. If not, we start the process again until a connected structure is achieved. We expect the degree distribution to be Poissonian in the resulting ER networks, which we verify in figure 2 by plotting the number of nodes with a given number of connections and comparing it to a Poisson distribution. Finally, we note that here most interactions are likely to be long range, in contrast to the NN networks described above.

### 3.3. Scale-free networks

We show here how we generate SF random networks that match the *N* and *K* values set by our NN network size. In the SF topology, linked agents are selected at random, as in the ER topology, but the resulting degree distribution must follow a power law of the form

3.1 $$n_{k}=C\;k^{b}.$$

Here, *n*_*k*_ is the number of agents with *k* connections, *C* is a constant prefactor, and *b* is the law’s exponent, which will always be negative [32,33].

In our finite and discrete system, *k* can only have integer values within a certain range, which we define to be from *k*_min_ to *k*_max_. We will set *k*_min_ = 2 in all our simulations, because it is the smallest number of connections for which an agent can be forced to align with the rest of the group in the AE model. Indeed, as we can intuitively see in its mechanical interpretation, any agent attached to the rest of the group through only one spring would be mechanically underconstrained (as it can rotate about its connection point) and thus able to point in any direction, without aligning to the collective motion. Note that we did not need to include the same restriction over *k*_min_ in the ER case, since the number of agents with less than two connections is negligible (less than 1%) for the corresponding degree distribution. At the other end of the distribution, we are restricted to always choose *k*_max_ ≤ *N* − 1, because we do not allow self-connections or more than one connection between two nodes. Finally, in order to generate SF networks with predetermined *N* and *K* values, the following two expressions must be satisfied

3.2 $$\sum_{k=k_{\min}}^{k_{\max}}n_{k}=N$$

and

3.3 $$\sum_{k=k_{\min}}^{k_{\max}}n_{k}\;k=K.$$

In principle, for any given exponent *b* we could find a solution for *C* and *k*_max_ that satisfies both expressions. In practice, however, the problem has additional constraints that make it slightly more complicated. Indeed, the *n*_*k*_ values resulting from equation (3.1) must be rounded to the nearest integer and they cannot be smaller than 1. We can thus only impose both constraints by modifying the degree distribution, redefining it as

3.4 $$n_{k}=\left\{\begin{array}{cc} \text{Round}(C\;k^{b}) & \text{if}\;C\;k^{b}\geq 1\;\\ 1\; & \text{if }C\;k^{b}<1, \end{array}\right.$$

where Round( · ) is a function that rounds to the nearest integer.

Our problem is now reduced to finding values for *C* and *k*_max_ that satisfy equations (3.2) and (3.3), using the *n*_*k*_ function defined in (3.4). Given that *k*_max_ and all *n*_*k*_ values must be integers, however, there is typically no exact solution for this system. We thus transform it into an optimization problem, finding the *C* and *k*_max_ values that minimize the difference between the left- and right-hand sides of equations (3.2) and (3.3). The corresponding objective function *J* is defined as the weighted sum of the squared error with respect to the target *N* and *K* values, which we define as *N*_tgt_ and *K*_tgt_. We thus have *J* = *W*_*N*_ (*N*_opt_ − *N*_tgt_)^2^ + *W*_*K*_ (*K*_opt_ − *K*_tgt_)^2^. Here, *N*_opt_ and *K*_opt_ are the optimizing variables while *W*_*N*_ and *W*_*K*_ are the manually tuned weights. To favour the convergence to the correct number of agents, we choose *W*_*N*_ = 10 and *W*_*K*_ = 1 (our results do not significantly depend on the exact choice of these values). Figure 2 presents an example of the resulting *n*_*k*_ values, labelled *rounded scale free*. Finally, we increase or decrease *k*_max_ as needed to exactly match the required total number of nodes *N* and then add or subtract a few connections by hand, to have *K* in total, so that all constraints are met. An example of the final *n*_*k*_ values is displayed in the curve labelled *modified scale free* in figure 2. It confirms that the final degree distribution still follows an approximate power law. We observe that it is almost identical to the *rounded scale free* distribution, showing that the required final manual adjustments are minimal. We also note that, for the *b* = −2 case displayed here, *k*_max_ is low enough for the distribution not to include a long flat region with *n*_*k*_ = 1 at high *k*-values. This is one of the reasons why we will set *b* = −2 in most simulations below.

Once the exact degree distribution is computed, we associate the corresponding number of links to each node and then connect all the links (of different nodes) at random, starting from the nodes with the highest degree. As in the ER case, we check that the resulting network is connected (which is almost always the case for SF networks) and, if not, start the process again.

### 3.4. Network superposition method

In the following sections, we will consider combinations of the network topologies defined above. To this end, we devised a *superposition protocol* that interpolates between an ordered NN network and either an ER or an SF random network, as a function of a *topological control parameter* *p* ∈ [0, 1]. For *p* = 0, we recover exactly the NN structure and for *p* = 1, one of the two random networks (ER or SF) with the same number of nodes and links. We will include these two extreme cases (which do not require using our superposition protocol) in our analysis below. In addition, by setting 0 < *p* < 1, we will be able to interpolate between NN and ER topologies or between NN and SF topologies.

Our superposition protocol is defined as follows. We begin by setting up an NN network of a given size, which determines the values of the total number of nodes *N* and total number of connections *K*. We then generate a realization of one of the two types of random networks (ER or SF) with *N* nodes and *K* links. Finally, we combine these networks by first deleting *p K* links from the NN network and (1 − *p*)*K* links from the ER or SF network, at random, and then superimposing the two resulting structures. By visualizing the resulting interaction network, as in figure 1, we verified that it remained connected for all intermediate *p*-values.

We note that our superposition protocol is similar to the rewiring method developed in [34] to generate small-world networks, but different in its details. In the small-world algorithm, a parameter *β*_sw_ (equivalent to our topological control parameter *p*) is used in a different way to interpolate between a regular lattice and a random network. Starting from a one-dimensional regular ring lattice where nearby nodes are connected, each link is rewired with probability *β*_sw_ to any other node in the ring, selected at random. For *β*_sw_ = 0, this approach also recovers the initial proximity network and for *β*_sw_ = 1 it generates a structure close to an ER random network. In our preliminary analyses, we verified that this method produces interaction networks on which our VN and AE simulations behave in the same qualitative way as described below. In what remains of this paper we will use our superposition approach, however, because it also allows us to interpolate between NN and random SF networks.

## 4. Simulations and results

We carried out multiple simulations of the VN and AE models (implemented as described in §2), using interaction topologies that interpolate either between NN and ER networks or between NN and SF networks (generated as detailed in §3).

In order to reduce the parameter space, we used the same AE model parameters in all our runs, setting *α* = 0.01, *β* = 0.12, *v*_0_ = 0.002 and *κ* = 5 while integrating equations (2.2) and (2.3) with time step Δ*t* = 0.1. These are the same parameters used in [11], which were shown to produce rapid and reliable self-organization into the aligned stated for systems with NN interaction topologies. We did not need to restrict the parameter space for the VN model, since equation (2.1) only depends on the noise intensity *η* and has no additional parameters.

We considered three different system sizes, all arranged in a square lattice configuration, as in figure 1: a small system consisting of 1024 agents (arranged in a 32 × 32 lattice) with 3906 connections, an intermediate system of 10 000 agents (100 × 100 lattice) with 39 402 connections, and a large system of 90 000 agents (300 × 300 lattice) with 358 202 connections. Given that reaching a well-converged statistically stationary state has different computational cost for different system sizes, models and network structures, we varied the number and duration of the runs accordingly. These additional simulation details are thus reported for each case in the corresponding figure caption. We include several examples of simulation videos and connectivity diagrams in the electronic supplementary materials.

In order to monitor the degree of order in the system, we used the standard polarization order parameter [10], given by

4.1 $$\psi =\frac{1}{N}∥\sum_{i=1}^{N}{\hat{n}}_{i}∥.$$

With this definition, if all agents are heading in a similar direction, we have *ψ* ≈ 1, and if they are disordered, we have *ψ* ≈ 0. For each model, topology and system size, we studied the degree of order achieved as a function of the angular noise *η*. We then identified the critical noise *η*_*c*_ of the order–disorder transition to analyse how it depends on the topology.

In all simulation results presented below, the swarm was initialized in the fully aligned state (*ψ* = 1). For the VN simulations, this choice of initial condition makes no difference in the resulting bifurcation diagrams, since these always produce a single branch, that is, a single stationary solution per noise value. For the AE simulations, however, it was shown in [11,12] that, in a regular lattice with NN interactions, there is a small region of bistability near the transition point where an ordered branch coexists with a disordered one. In the AE case with long-range interactions studied here, we found that this bistability region displays a more complex behaviour, since it can extend to *η* = 0 for topologies with a certain level of randomness. This implies that, even without noise, the system never reaches an aligned self-organized state, remaining instead trapped in some kind of chaotic dynamics. In our preliminary analysis, we found that the presence of this disordered branch for *η* < *η*_*c*_ has a complex dependence on the topological structure. Its understanding would thus require an extensive sampling of the possible random interaction topologies, which is beyond the scope of this paper. We will therefore only search here for the upper branch of each bifurcation diagram (i.e. for the ordered state, in regions where the system is bistable), which is why we chose to initialize all simulations in the fully aligned state. A detailed study of the relationship between the interaction topology and the disordered branch will be left for future work.

### 4.1. Bifurcation diagrams

We begin by presenting bifurcation diagrams that display the order parameter *ψ* as a function of noise intensity *η* for different topologies, first in simulations of the VN model and then of the AE model. Each point in these bifurcation diagrams is the result of averaging the mean *ψ* values of multiple runs. For *p* > 0, each one of these runs was performed on a different random superposition of an NN network and a random ER or SF interaction network, which remained fixed throughout the simulation. The degree distributions of all the random SF networks considered in this section were generated using a *b* = −2 exponent.

#### 4.1.1. Vicsek-Network model bifurcation diagrams

**Figure 3.**
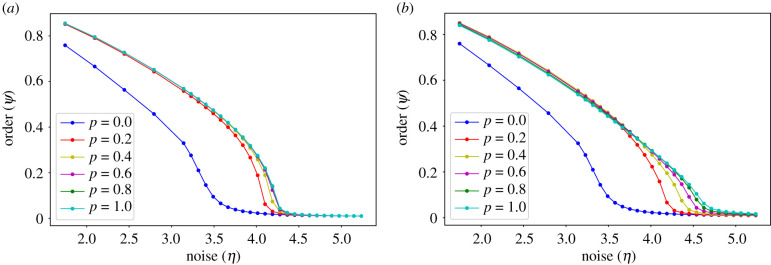
Bifurcation diagrams of the VN model with different interaction topologies, ranging from NN (*p* = 0.0) to ER (*p* = 1.0) networks in panel (*a*) and from NN (*p* = 0.0) to SF (*p* = 1.0) networks with *b* = −2 in panel (*b*). The transition appears as continuous for all cases. A larger fraction of random long-range connections (either ER or SF) increases the critical noise. Each point results from eight runs (each one with a different random superposition of an NN and a random ER or SF network) of 5 × 10^5^ time steps per noise value, for an intermediate system size (*N* = 100 × 100).

Figure 3*a* displays the bifurcation diagrams for the VN model, with interaction topologies interpolating between NN and random ER network structures, whereas figure 3*b* displays the corresponding diagrams with topologies interpolating between NN and random SF networks. The interpolating parameter *p* controls the topology, with *p* = 0 corresponding to the NN case and *p* = 1 to the random case.

For *p* = 0, the same curve is displayed in both plots. It appears to show a continuous transition from an aligned to a disordered state. We note, however, that this must be a finite-size effect, since it is well known that (in agreement with the Mermin–Wagner theorem [35]) the VN model with fixed NN interactions cannot present long-range order for any non-vanishing noise *η* in the ‘thermodynamic limit’ of infinite system size. Indeed, for *η* > 0 and *N* → ∞, the standard Vicsek model can only reach a stationary state with *ψ* > 0 if the interacting agents change throughout the dynamics [10] or if the interactions are non-local [26]. Despite its finite-size nature, we display this typical transition curve for the system size considered, in order to compare it to the curves obtained with non-local connections.

The *p* = 1 curves in both panels of figure 3 also correspond to well-studied cases. The VN case is equivalent to the mean-field system solved in [26,36], whereas the SF case is equivalent to that solved in [37]. This is because, as the system grows, an interaction network that connects to a given number of fixed randomly selected agents becomes equivalent to randomly sampling the states of the same number of agents, as in a mean-field approximation. Note that these analyses predict a standard mean-field critical exponent of 1/2 for the ER case and a non-standard critical exponent, which departs from the standard mean-field universality class and depends on *b*, for the SF case [28]. The critical exponents that we observe in figure 3 for the *p* = 1 case appear to be consistent with these results.

The curves with intermediate values of *p* in figure 3 show that a few random non-local interactions (with ER or SF topology) are enough to significantly increase the critical noise, and therefore the resilience to noise of the system. This is not surprising, since we typically expect a small fraction of long-range interactions to strongly increase system integration, and thus resilience to noise, as it has been shown for small-world networks [38]. In addition, the plots show that long-range links with SF topology increase more effectively the resilience to noise than those with ER topology. This is also consistent with expectations, since in SF networks the mean distance between nodes is significantly reduced [39], which will improve the convergence of the distributed average consensus protocol that underlies the VN model, thus producing higher critical noise values.

#### 4.1.2. Active-Elastic model bifurcation diagrams

**Figure 4.**
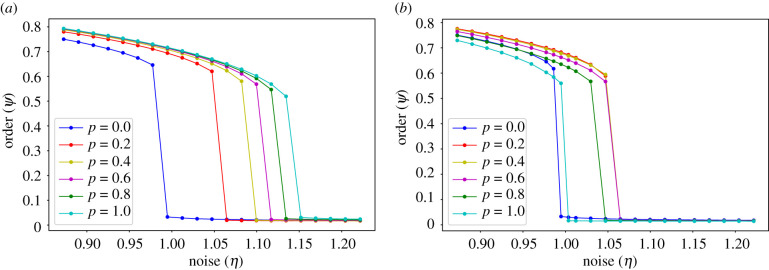
Bifurcation diagrams of the AE model with different interaction topologies, ranging from NN (*p* = 0.0) to ER (*p* = 1.0) networks in panel (*a*), and from NN (*p* = 0.0) to SF (*p* = 1.0) networks with *b* = −2 in panel (*b*). All transitions appear to be discontinuous. For each one, we display here only the upper solution branch. In contrast to the VN case presented in figure 3, the critical noise only increases monotonically with the fraction of random long-range connections in panel (*a*), while it decreases for *p* > 0.6 in panel (*b*). Each point results from 40 runs (each one with a different random superposition of an NN and a random ER or SF network) of 10^6^ time steps per noise value, for an intermediate system size (*N* = 100 × 100).

Figure 4*a* presents the bifurcation diagrams for the AE model with topologies interpolating between NN and random ER networks, whereas figure 4*b* displays the corresponding diagrams with topologies interpolating between NN and random SF networks. Here again, *p* is the interpolating parameter.

In this model, the transition appears to be first-order for all values of *p*. This is consistent with previous results obtained in the *p* = 0 case [11], where it was shown that the ordered and disordered branches can co-exist for a range of *η*-values close to the critical noise. As explained at the beginning of this section, we only find here the ordered branch (in the bistable region), because all simulations were started from a fully aligned (*ψ* = 1) initial condition. When we include a small fraction of long-range random connections, for *p* = 0.2, we observe a significant increase in the resilience to noise. In the ER case, the critical noise then continues to increase with *p*, as in the previously presented VN case, which is consistent with the aforementioned expectation that more long-range interactions favour system integration. In the SF case (figure 4*b*), however, the critical *η* starts decreasing for *p* > ∼0.6, so resilience to noise is maximized for intermediate values of *p*. This is surprising, since it implies that including more long-range interactions can hinder system cohesion. We also observe that the fully random SF topology (*p* = 1) displays an even lower critical noise value, which contradicts the common notion that SF networks should improve system integration by reducing the mean distance between nodes [39].

### 4.2. Critical noise as a function of topological structure

We present here the critical noise value *η*_*c*_, as a function of the topological control parameter *p*, for all the bifurcation diagrams presented above and for other system sizes. In order to do this, we must first define objective criteria for computing the transition point from our numerical data.

For the VN model, given that the transition appears as continuous, we used a standard method for detecting the critical noise in second-order phase transitions. We thus identified *η*_*c*_ as the point where the variance of the order parameter *ψ* is maximized. To interpolate between the discrete simulated values of *η*, we selected the *η* value with the highest $\text{Var}(\psi_{\eta })$ (the variance of the order parameter over all runs performed with noise level *η*) and the two adjacent *η*-values. We then computed *η*_*c*_ as the location of the maximum of a quadratic curve that passes through the three corresponding $\left(\eta ,\text{Var}(\psi_{\eta })\right)$ points. For the AE model, since in this case the transition is discontinuous, we defined *η*_*c*_ as the point where the ordered solution ceases to exist. The critical noise level was thus computed as the midpoint between the highest *η* value at which we identified an ordered solution and the next tested value of *η*, for which no ordered stationary state could be found. Using these criteria, we computed the critical noise *η*_*c*_ for three different system sizes in each model and topology considered.

**Figure 5.**
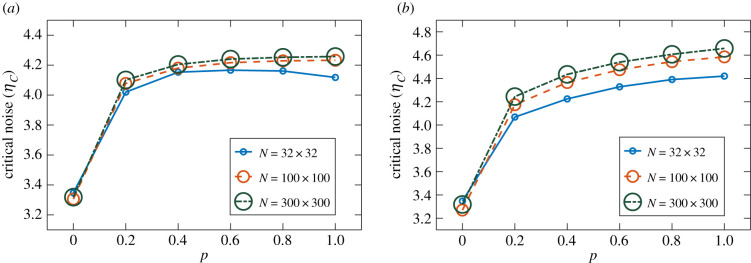
Critical noise *η*_*c*_ as a function of the topological control parameter *p* for the VN model with different system sizes. The parameter *p* interpolates between NN and ER random networks in (*a*), and between NN and SF (with *b* = −2) random networks in (*b*). As the fraction of random, long-range connections is increased, the critical noise increases in both cases. Small system (*N* = 32 × 32): 40 runs of 2 × 10^5^ time steps per noise value. Intermediate system (*N* = 100 × 100): eight runs of 5 × 10^5^ time steps per noise value. Large system (*N* = 300 × 300): eight runs of 10^5^ time steps per noise value.

Figure 5 displays *η*_*c*_ as a function of *p* for the VN model, with *p* interpolating between NN and ER networks in panel (*a*) and between NN and SF networks in panel (*b*). As previously discussed, we observe the same behaviour in both cases: the resilience to noise increases with *p* (i.e. with the fraction of random connections). The only exception is the *N* = 32 × 32 case in panel (*a*), where strong finite size effects appear to reduce the resilience to noise as *p* approaches 1. In both cases, we note that the benefit of adding more random links starts saturating for larger *p*.

**Figure 6.**
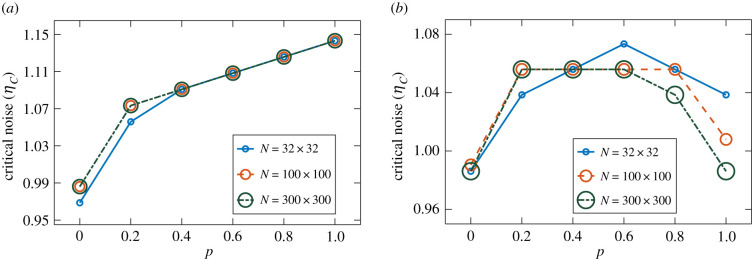
Critical noise *η*_*c*_ as a function of the topological control parameter *p* for the AE model with different system sizes. The parameter *p* interpolates between NN and ER random networks in (*a*), and between NN and SF (with *b* = −2) random networks in (*b*). The ER case displays higher *η*_*c*_ for higher *p*-values, as in figure 5. In the SF case, however, the maximum *η*_*c*_ is reached here at intermediate *p* values. Small system (32 × 32): 40 runs of 10^6^ time steps per noise value. Intermediate system (100 × 100): 40 runs of 10^6^ time steps per noise value. Large system (300 × 300): eight runs of 5 × 10^5^ time steps per noise value.

Figure 6 shows *η*_*c*_ as a function of *p* for the AE model. When *p* interpolates between NN and ER networks, as shown in panel (*a*), the resilience to noise increases with *p* in a way similar to the VN case. We note, however, that *η*_*c*_ appears to grow linearly with *p* for *p* ≥ 0.4 in all system sizes, without starting to saturate as in the VN case. When *p* interpolates between NN and SF networks, in panel (*b*), we can clearly see the previously described surprising behaviour; the resilience to noise increases initially with *p*, but then decreases as *p* approaches 1. This reduction of the critical noise for larger *p*-values is seen to depend on system size, with *η*_*c*_ becoming smaller at *p* = 1 in bigger systems.

The reduced resilience to noise for a larger fraction of random SF connections observed above in the AE model appears to be a consequence of the overabundance of nodes with low degree. This is because, as we will discuss in §5, nodes with low degree are more easily excitable by noise, since they represent weakly coupled components of the elastic network that mediates the interactions in the AE model. This is also consistent with the decrease of resilience in larger systems, since the fraction of nodes with few connections increases with *N*. In order to test this hypothesis, we will analyse below the relationship between critical noise and the exponent of the degree distribution in SF interaction networks.

### 4.3. Critical noise as a function of scale-free exponent

We consider here simulations of the AE model interacting through fully random (*p* = 1) SF topologies, analysing the relationship between the critical noise and the exponent *b* of the degree distribution in equation (3.4).

**Figure 7.**
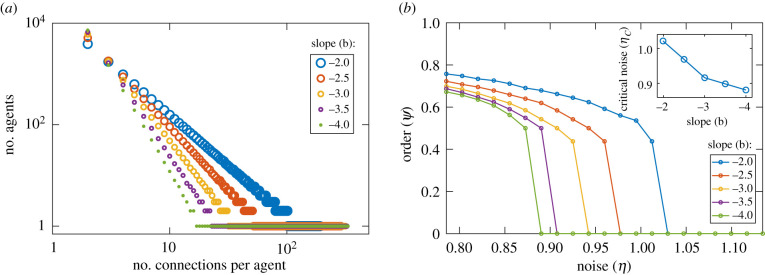
Simulation details and results of our AE model analysis of the relationship between the critical noise and the SF interaction network exponent. Panel (*a*) presents the different degree distributions, with various *b* exponents, that were implemented in the fully random (*p* = 1) SF interaction networks considered. Each *b* corresponds to a different slope in this log–log plot. Panel (*b*) displays the resulting bifurcation diagrams for each of these distributions. The inset shows the critical noise as a function of *b*. We observe that *η*_*c*_ decreases for steeper distribution slopes. All simulations were carried out in an intermediate size system (*N* = 100 × 100), performing 16 runs of 2 × 10^5^ time steps per *b* value.

Figure 7*a* shows the different degree distributions that we implemented in our intermediate (*N* = 100 × 100) system size, following the procedure detailed in §3.3. Note that, in order to keep *N* and *K* constant, we must increase *k*_max_ for higher values of |*b*| , which extends the *n*_*k*_ = 1 region where the distribution is flat.

For each exponent *b*, we then performed multiple simulations of the AE model, using different realizations of an SF interaction network with the corresponding degree distribution. Figure 7*b* presents the resulting bifurcation diagrams. The inset displays the respective critical noise values as a function of *b*. For all SF exponents considered, we find a discontinuous order–disorder transition, as in the previously explored *b* = −2 case. The figure shows that *η*_*c*_ is reduced as the slope of the distribution becomes steeper. This implies that the resilience to noise decreases as *b* becomes more negative, that is, as the fraction of nodes with a low number of connections is increased, in agreement with our hypothesis in the previous subsection.

## 5. Discussion

The results presented above show that the structure of the interaction network affects very differently the resilience to noise of the ordered state in the VN model and the AE model. In the VN case, a larger fraction of random links with either Poissonian or power-law degree distribution always increases the critical noise. In the AE case, we obtain similar results only when interpolating between NN and random ER network topologies. When interpolating instead between NN and random SF networks, a larger fraction of random links can reduce the critical noise. As mentioned above, this is contrary to the common intuition that more long-range connections tend to improve the collective behaviour of networked systems by decreasing the mean topological distance between nodes, which improves the system-wide propagation of information. This is believed to be especially true when these connections follow an SF topology, because this connectivity further reduces the mean distance between nodes.

A heuristic explanation of this phenomenon can be deduced from the difference in the self-organization mechanism that leads to collective motion in each model. The self-organizing dynamics of the VN model is based on a distributed average consensus process, where local orientation averages of the consensus variable propagate directly through the network. In the AE model, however, only the positional information is propagated through the network, while the consensus variable is still the orientation. This suggests that the self-organizing mechanism for the AE model must be different. Indeed, as detailed in [11,12], the AE model self-organizes by focusing the self-propulsion energy into low-energy modes, through a combination of a well-known property of all elastic systems and the coupling (imposed by its dynamical equations) between the elastic forces and the individual heading directions. It is well known that higher energy modes dampen at a faster rate than slower ones in all elastic systems, because their higher rigidity produces higher oscillation frequencies that dissipate faster. The AE model is an *active* elastic system, however, where each agent is also continuously injecting energy at the individual level through its self-propulsion term, so motion cannot dampen out. Instead, the elastic forces will tend to steer agents away from the higher modes, which require more energy to excite. The self-propulsion energy will thus be channelled to lower and lower modes, until the first (rotational) mode or the zero (translational) mode is reached and collective motion is achieved.

With this explanation, we can understand why SF networks can lead here to weaker ordered states. Previous works had only considered homogeneous elastic systems with NN interactions, in which low-energy elastic modes correspond to large scales of coherent motion. In complex, networked elastic systems, however, this correspondence does not hold, since there can be highly localized and disordered low-energy modes that do not correspond to any large-scale collective dynamics. In particular, our SF networks will have an overabundance of agents with few connections, and therefore low elastic constraints, which will result in multiple disordered low-energy modes. There will be, for example, a majority of agents with only two links, which can be easily excited at very low energy levels.

The discussion above helps us understand why disordered modes can be easily excited by noise in the AE model with SF interactions. This in turn justifies the lower critical noise that we observe for the AE model as more random SF interaction links are included, and when considering SF interaction networks that are larger or have steeper degree distributions.

## 6. Conclusion

In this paper, we have explored for the first time the relationship between the interaction topology and the self-organizing properties of two different types of models of collective motion: one velocity-based and one position-based. We found that the interaction network can have very different effects on the critical noise of their corresponding order–disorder transitions. In the position-based model, in particular, we observed that an interaction network with power-law degree distribution can hinder self-organization, when compared to a proximity network, despite having a large fraction of long-range connections.

These results could have implications in various fields. In the study of biological collective motion, they imply that the structure of the interaction network may play a fundamental role in determining how different animal groups self-organize, which could be tested experimentally. In swarm robotics, where we can often choose which agents interact to achieve the desired collective dynamics, these results could help guide the design of effective interaction networks for various types of decentralized motion control algorithms.

Finally, the strong differences displayed by the two models when interacting through various network structures suggest that they may belong to two distinct classes of self-organizing systems: one for which the ordered state is always favoured by long-range interactions and one where it depends on more subtle structural properties of the interaction network. In future work, it would be interesting to search for other examples of models that belong to each class.

## Supplementary Material

README.txt

## Supplementary Material

BifurcationDiagram Simulations AE_ER.png

## Supplementary Material

BifurcationDiagram Simulations AE_SF.png

## Supplementary Material

BifurcationDiagram Simulations VN_ER.png

## Supplementary Material

BifurcationDiagram Simulations VN_SF.png

## Supplementary Material

ConnectivityDiagram_NN2ER_p02.png

## Supplementary Material

ConnectivityDiagram_NN2ER_p10.png

## Supplementary Material

ConnectivityDiagram_NN2SF_p02.png

## Supplementary Material

ConnectivityDiagram_NN2SF_p10.png

## Supplementary Material

Simulation AE ER_p02 eta57.avi

## Supplementary Material

Simulation AE ER_p02 eta61.avi

## Supplementary Material

Simulation AE ER_p10 eta63.avi

## Supplementary Material

Simulation AE ER_p10 eta67.avi

## Supplementary Material

Simulation AE SF_p02 eta57.avi

## Supplementary Material

Simulation AE SF_p02 eta61.avi

## Supplementary Material

Simulation AE SF_p10 eta55.avi

## Supplementary Material

Simulation VN SF_p10 eta250.avi

## Supplementary Material

Simulation VN SF_p02 eta180.avi

## Supplementary Material

Simulation VN SF_p02 eta250.avi

## Supplementary Material

Simulation VN SF_p10 eta180.avi

## Supplementary Material

Simulation AE SF_p10 eta59.avi

## Supplementary Material

Simulation VN ER_p02 eta180.avi

## Supplementary Material

Simulation VN ER_p02 eta250.avi

## Supplementary Material

Simulation VN ER_p10 eta180.avi

## Supplementary Material

Simulation VN ER_p10 eta250.avi

## Data accessibility

This article has no additional data.
