# Supplementary figures and images for: Interaction network effects on position- and velocity-based models of collective motion
Source: J R Soc Interface. 2020 Aug 19;17(169):20200165. doi: 10.1098/rsif.2020.0165 (PMC7482575; doi:10.1098/rsif.2020.0165)

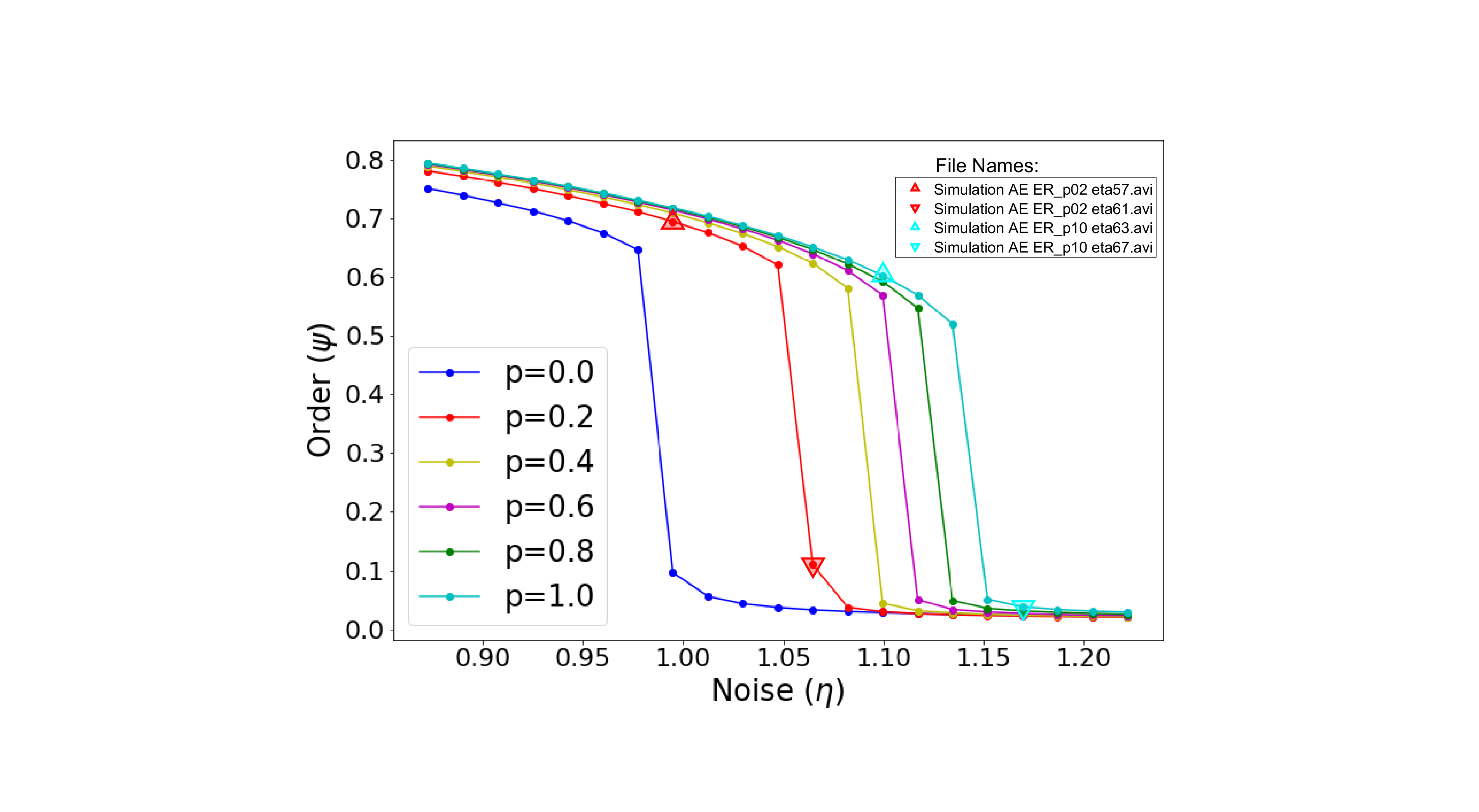

Supplement: BifurcationDiagram Simulations AE_ER.png [file rsif20200165supp2.png]

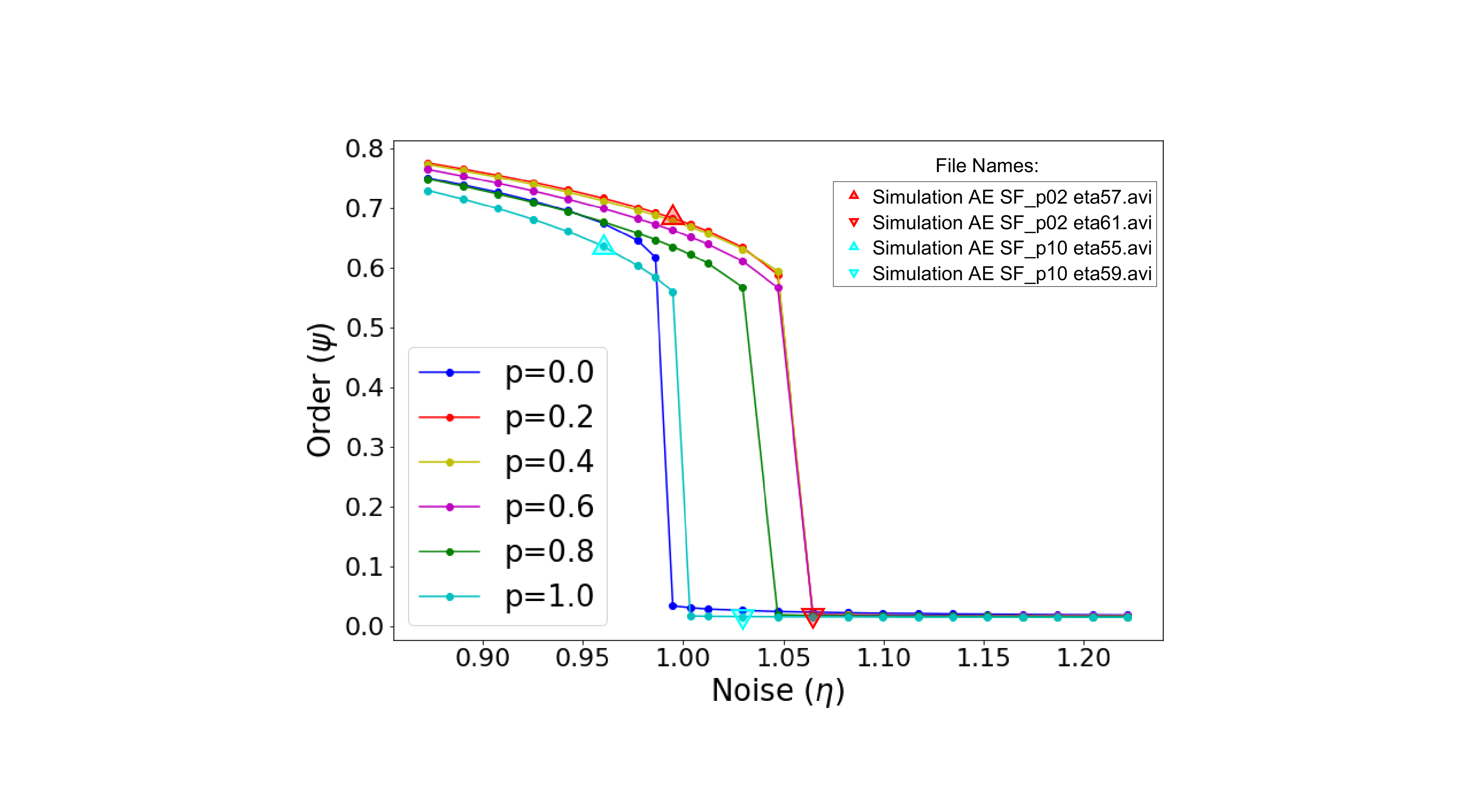

Supplement: BifurcationDiagram Simulations AE_SF.png [file rsif20200165supp3.png]

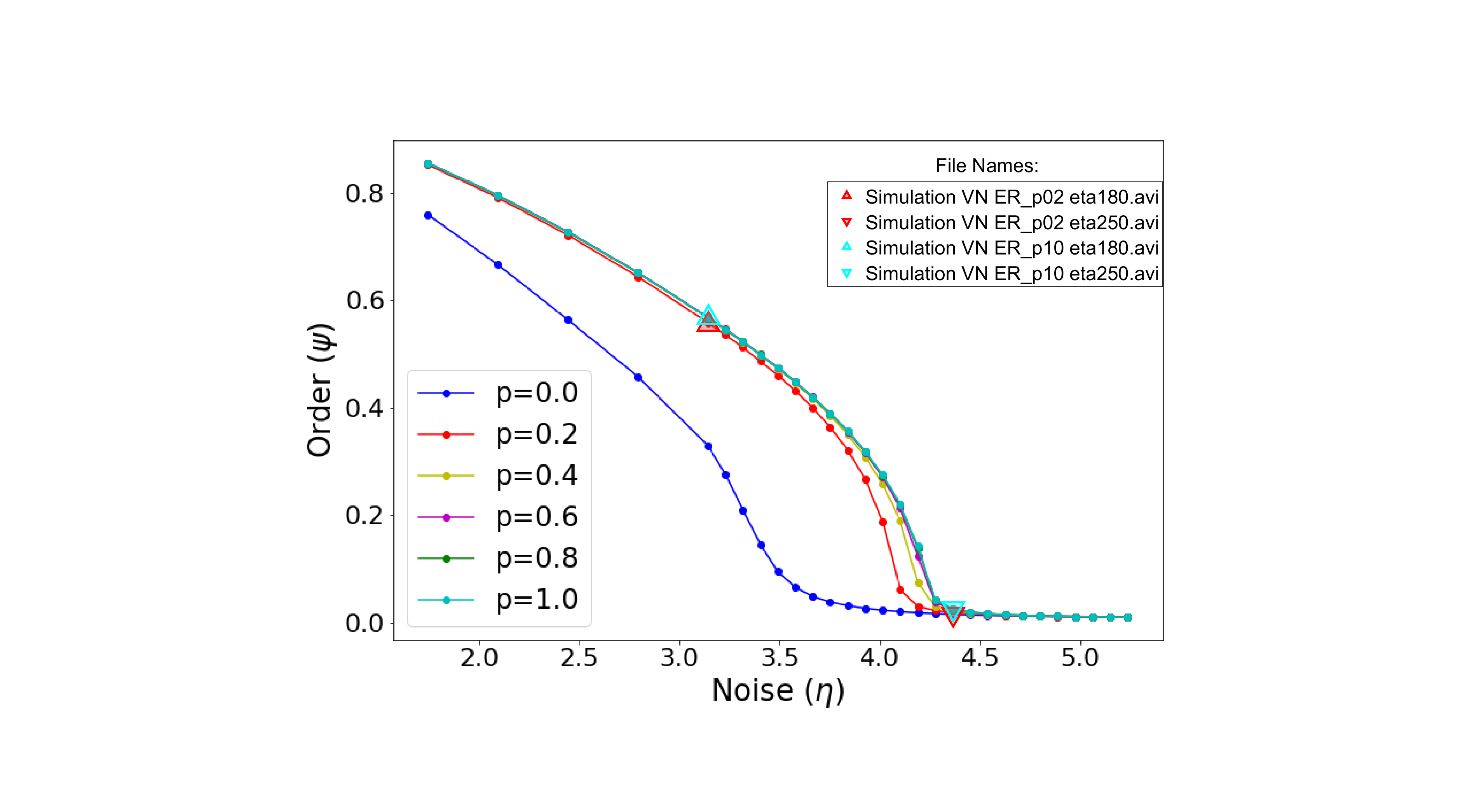

Supplement: BifurcationDiagram Simulations VN_ER.png [file rsif20200165supp4.png]

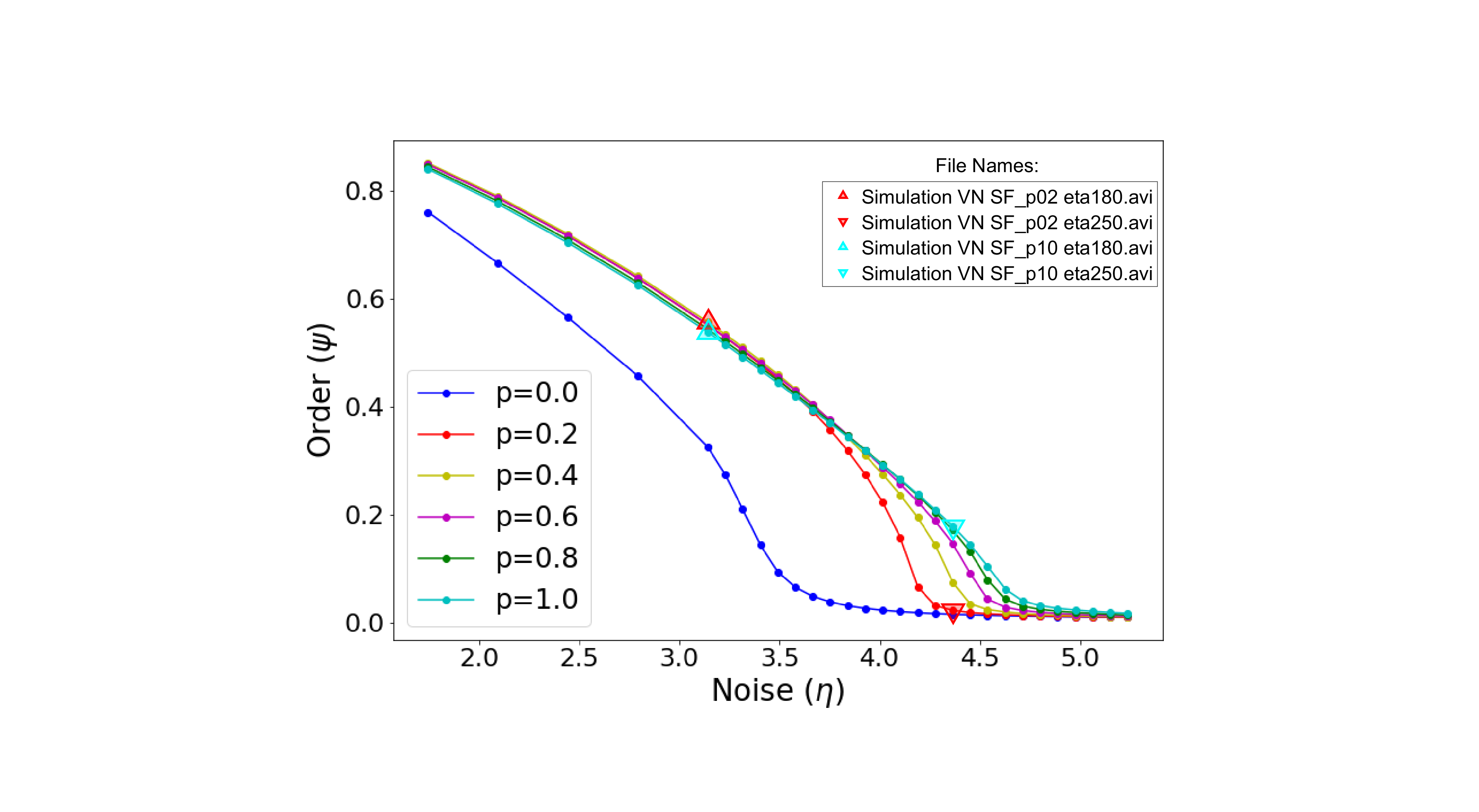

Supplement: BifurcationDiagram Simulations VN_SF.png [file rsif20200165supp5.png]

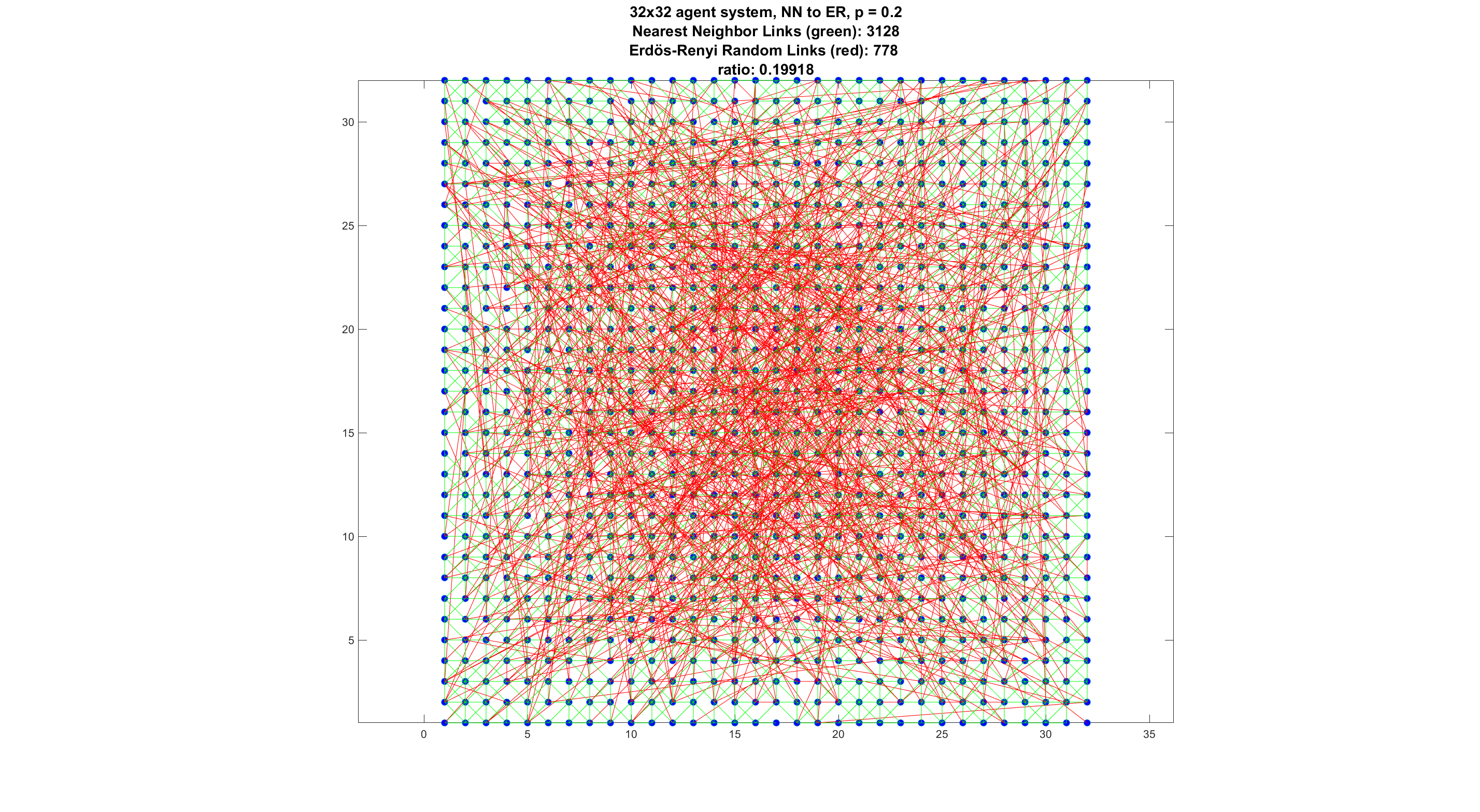

Supplement: ConnectivityDiagram_NN2ER_p02.png [file rsif20200165supp6.png]

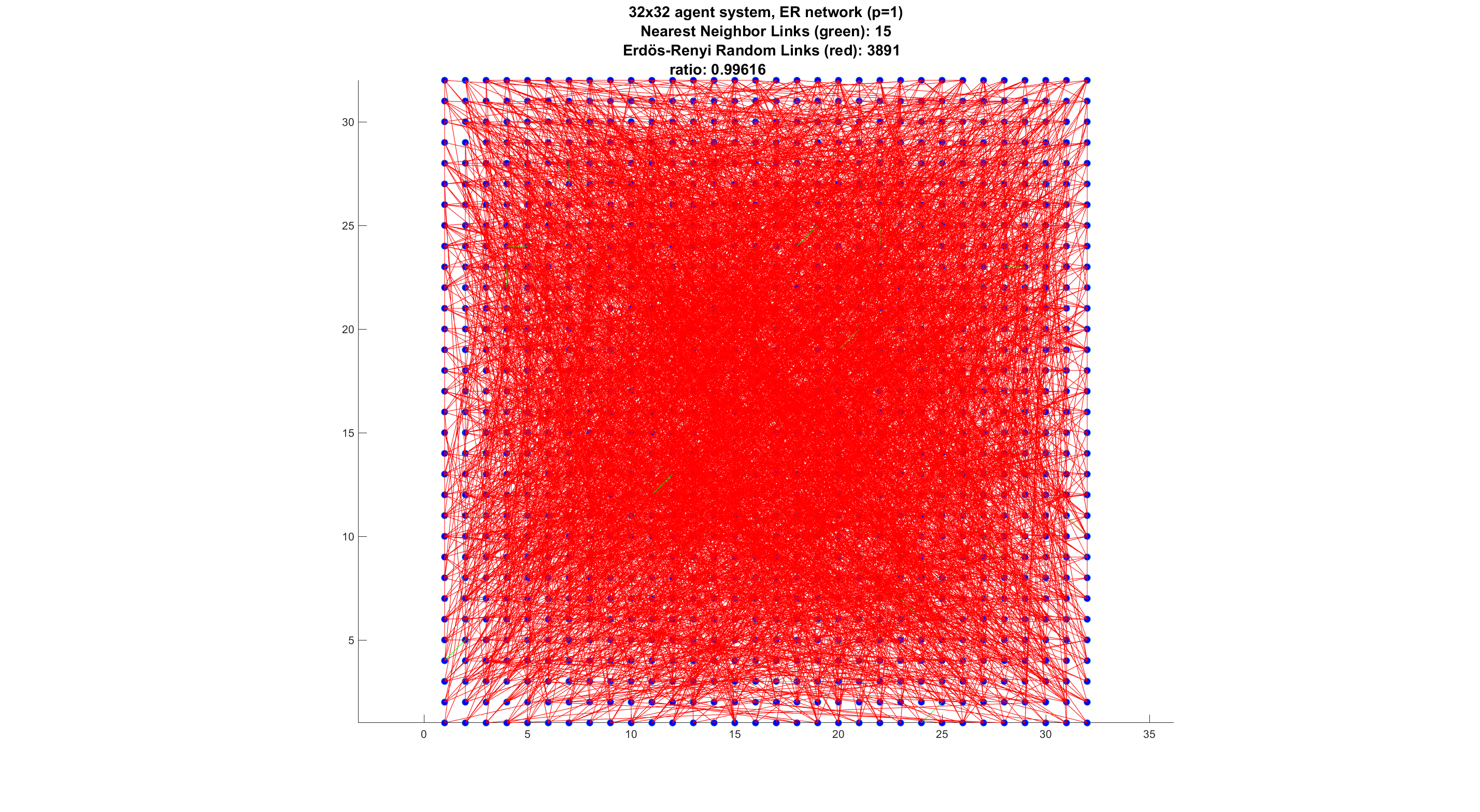

Supplement: ConnectivityDiagram_NN2ER_p10.png [file rsif20200165supp7.png]

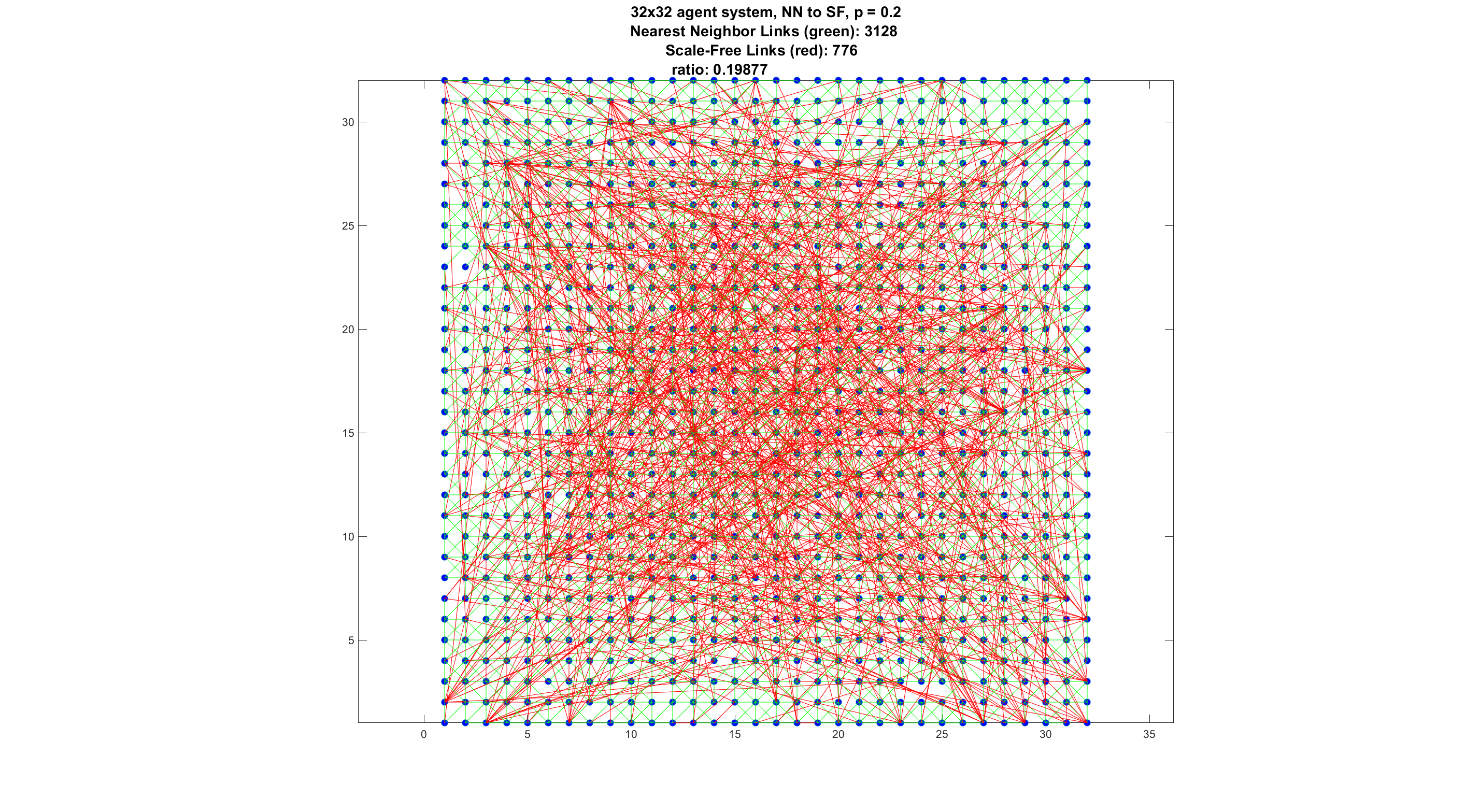

Supplement: ConnectivityDiagram_NN2SF_p02.png [file rsif20200165supp8.png]

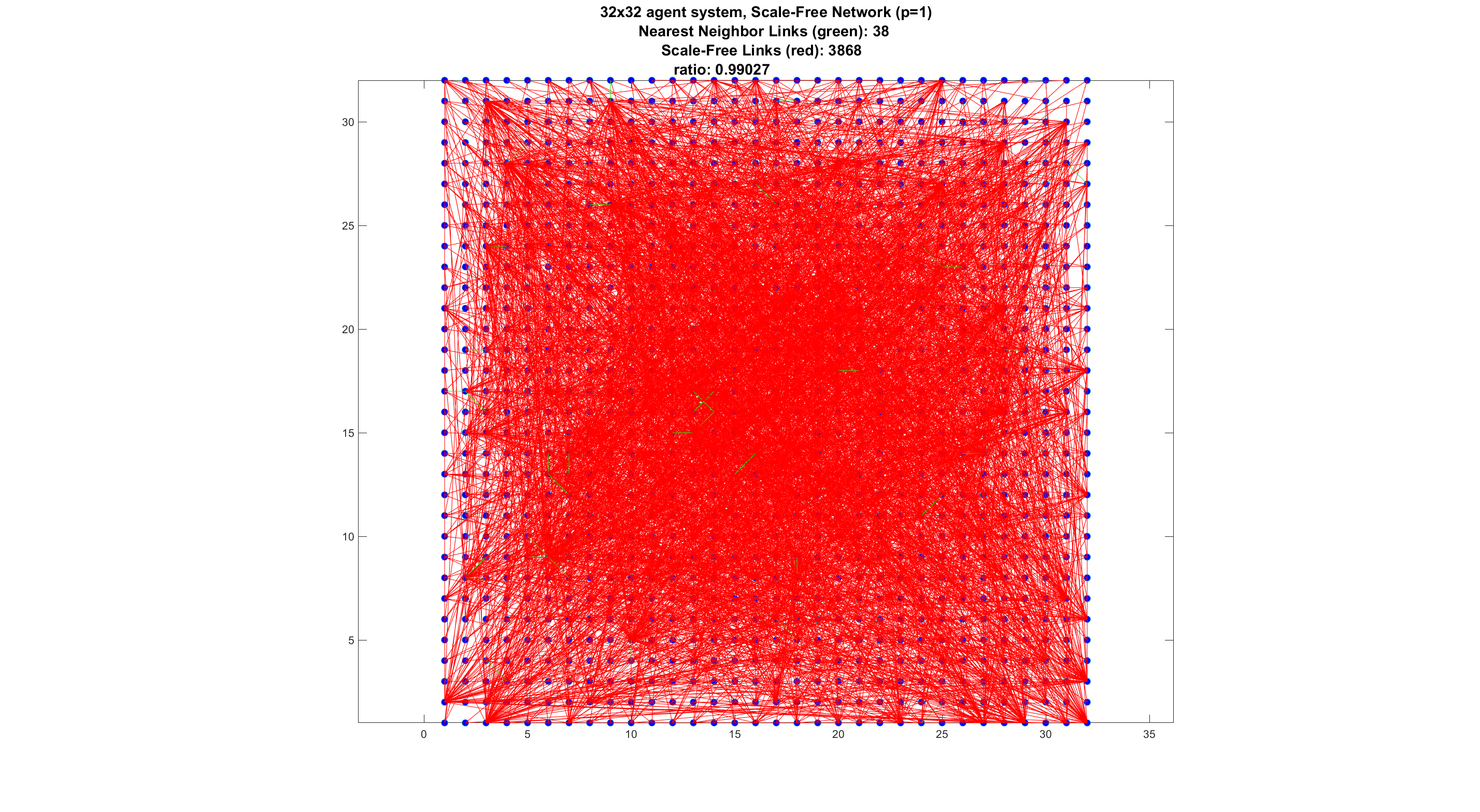

Supplement: ConnectivityDiagram_NN2SF_p10.png [file rsif20200165supp9.png]
